# FGF21 as Modulator of Metabolism in Health and Disease

**DOI:** 10.3389/fphys.2019.00419

**Published:** 2019-04-17

**Authors:** Caterina Tezze, Vanina Romanello, Marco Sandri

**Affiliations:** ^1^Veneto Institute of Molecular Medicine, Padua, Italy; ^2^Department of Biomedical Science, University of Padua, Padua, Italy; ^3^Department of Medicine, McGill University, Montreal, QC, Canada; ^4^Department of Biomedical Science, Myology Center, University of Padua, Padua, Italy

**Keywords:** FGF21, skeletal muscle, metabolism, regulation, myokine, cytokine, mitochondria

## Abstract

Fibroblast growth factor 21 (FGF21) is a hormone that regulates important metabolic pathways. FGF21 is expressed in several metabolically active organs and interacts with different tissues. The FGF21 function is complicated and well debated due to its different sites of production and actions. Striated muscles are plastic tissues that undergo adaptive changes within their structural and functional properties in order to meet their different stresses, recently, they have been found to be an important source of FGF21. The FGF21 expression and secretion from skeletal muscles happen in both mouse and in humans during their different physiological and pathological conditions, including exercise and mitochondrial dysfunction. In this review, we will discuss the recent findings that identify FG21 as beneficial and/or detrimental cytokine interacting as an autocrine or endocrine in order to modulate cellular function, metabolism, and senescence.

## Introduction

Nearly 40–50% of total body mass in non-obese mammals is composed of skeletal muscle. Striated muscles are plastic tissues that undergo adaptive changes in their structural or functional properties in order to meet new challenges. Skeletal muscle tissues have generally been considered to be pure locomotor organs, but in the 2000s it was recognized a secretory function for these muscles. In 2003 it was proposed that IL-6 and other cytokines were induced and secreted by skeletal muscles into their circulation during physical activity. In consideration of these findings, the muscle-secreted molecules were named “myokines.” Increasing evidence underlines that skeletal muscle secretes a wide variety of molecules like cytokines, miRNA, exosomes, mtDNA during exercise, but also in different acquired and inherited diseases (Inagaki et al., 2007; Alipoor et al., 2016; Hoffmann and Weigert, 2017; Delezie and Handschin, 2018; Safdar and Tarnopolsky, 2018). Thus, skeletal muscles are understood to be a source of myokines, metabolites, and muscle-derived molecules. They mediate communication between distant organs to adapt whole body metabolism to nutritional and environmental pressures (Rai and Demontis, 2016; Whitham and Febbraio, 2016). This systemic regulation helps to explain why physical activity, and thereby muscle contraction, elicits several beneficial effects in a variety of diseases. However, the nature and the function of most of the myokines and muscle-derived molecules are still unclear. In this review, we will focus on fibroblast growth factor 21 (FGF21) and its role as a “myokine.” Several studies have proven that FGF21 stimulates the oxidation of fatty acids, the production of ketone bodies, and the inhibition of lipogenesis (Fisher and Maratos-Flier, 2016; Staiger et al., 2017). Therefore, the finding that FGF21regulates glucose-lipid metabolism has made it a promising therapeutic target for metabolic disease. However, some studies have shown that administrating FGF21 prevents diet-induced obesity and insulin resistance in mice and humans (Fisher and Maratos-Flier, 2016; Staiger et al., 2017). There is a paradoxically positive correlation with elevated serum FGF21 levels and metabolic disorders like obesity, diabetes, mitochondrial diseases, and aging (Staiger et al., 2017; Tezze et al., 2017). Interestingly, all these conditions have muscle loss as a common factor. In addition, several reports have indicated that a pathophysiological role for FGF21 includes:

(a) the promotion of muscle atrophy (Oost et al., 2019), bone loss and reduced bone mineral density (Wei et al., 2012; Fazeli et al., 2015).
(b) that it acts as a stress-induced myokine, which is released during starvation, ER stress, and mitochondrial dysfunction (Izumiya et al., 2008; Keipert et al., 2013; Pereira et al., 2017; Tezze et al., 2017; Rodríguez-Nuevo et al., 2018).

Thus, FGF21 is a powerful and debatable hormone that belongs to the list of factors that controls energy homeostasis and metabolism. As described below, the beneficial and/or detrimental actions of FGF21 are affected by multiple variables including tissue source, serum concentration, animal age, and the presence of synergizing factors.

## FGF21 and Metabolism

The fibroblast growth factor (FGF) family is a group of multifunctional signaling molecules that have a wide variety of functions. The family comprises of 22 related proteins, each grouped into subfamilies which are based on genetic and functional similarity. In Nishimura et al. (2000) the *Fgf21* gene was identified in mouse embryos by RT-PCR. Making it the 21st discovered *Fgf* gene, which was later to be included into the FGF19 subfamily (also called endocrine FGF), together with *Fgf15* (mouse ortholog of human FGF19) and *Fgf23*. FGF21 function remained completely unknown until 2005 when it was proposed to be a novel metabolic regulator and a potential anti-diabetic drug (Kharitonenkov et al., 2005). Kharitonenkov et al. (2005) demonstrated that the administration of FGF21 reduced plasma glucose and triglycerides to an almost normal level in both ob/ob and db/db mice. These effects persisted for up to 24 h after which the last FGF21 was administrated. Over the following years, several groups investigated the metabolic role of FGF21 in mice. Their results showed that FGF21 mediates the adaptive starvation response to induce ketogenesis, gluconeogenesis, lipolysis, and lipid β-oxidation (Inagaki et al., 2007; Izumiya et al., 2008). In line with Kharitonenkov results, FGF21 treatment improved metabolic parameters in obese and diabetic animal models (Coskun et al., 2008; Xu et al., 2009). In humans, FGF21 is a starvation-induced protein that is elevated after 7 days of fasting and regulates the utilization of fuel to adapt metabolism in the late phase of the absence of nutrients (Fazeli et al., 2015). FGF21 is expressed in several tissues such as liver (Nishimura et al., 2000), adipocytes (Zhang et al., 2008), pancreas (Johnson et al., 2009), and brain where it passes the blood-brain barrier (Hsuchou et al., 2007). Other studies reported the expression of FGF21 mRNA in testes, gastrointestinal tract, brain, skeletal muscle, brown adipose tissue (BAT), and heart (Patel et al., 2015). Based on its role in glucose and lipid metabolism and its wide tissue expression, it was hypothesized that FGF21 is a fasting-adaptation hormone in rodents (Fazeli et al., 2015). However, it was recently demonstrated that FGF21-knockout mice do not exhibit an impaired response to fasting (Antonellis et al., 2016) suggesting that FGF21 is not required for the physiological response to low nutrients.

## FGF21 Expression and Regulation

The FGF21 regulation is complicated because of its different tissue production and action. The liver is considered to be the main site of FGF21 production (Badman et al., 2007; Inagaki et al., 2007). Badman and Inagaki independently showed a cross-talk between FGF21 and PPARα signaling in the liver during fasting. FGF21 expression was increased in mice that were either fed with a ketogenic diet or through fasting and in these conditions, PPARα was recruited in the different regions of the *Fgf21* promoter (Badman et al., 2007; Inagaki et al., 2007). However, this finding was not confirmed in other metabolic conditions in which FGF21 was induced, suggesting that other transcription factors might also regulate hepatic FGF21 expression. Indeed, *Fgf21* was found to be under the regulation of the Unfolding Protein Response (UPR) in hepatocytes (Schaap et al., 2013; Kim et al., 2015). Kim et al. (2015) started from the observation that FGF21 increased in the liver of patients who were affected by steatosis, and mouse models of obesity or non-alcoholic fatty liver disease (NAFLD) while at the same time, observing that an ER stress became triggered. They found that FGF21 expression was dependent from PKR-like ER kinase (PERK)–eukaryotic initiation factor 2α (eIF2α)–activating transcription factor 4 (ATF4) pathways both, *in vitro* and *in vivo* (Kim et al., 2015). Moreover, FGF21-null mice displayed induction of ER stress markers in genes and observed more lipid accumulation in the liver after tunicamycin treatment, an inhibitor of glycoprotein biosynthesis that promotes ER stress (Kim et al., 2015). Several studies have suggested that other transcription factors are involved in the regulation of hepatic FGF21 expression. Adams et al. (2010) reported that thyroid hormone receptor β, which mediates the action of tri-iodothyronine in the liver, stimulates lipolysis, and hepatic fatty acid oxidation via FGF21 induction. Moreover, several researchers have speculated that FGF21 expression is regulated by RARβ (Wang et al., 2010, p. 21; Berry et al., 2012; Tsuchiya et al., 2012; Li et al., 2013). This is because the metabolic effect of the RARb ligand, retinoic acid, on body weight loss and glucose/lipid metabolism are similar to FGF21, this has been recently demonstrated *in vitro* in C2C12 (Hirai et al., 2019) but has not yet been confirmed *in vivo*. Retinoic acid receptor-related orphan receptor (ROR) α has been implicated in various physiological functions, including the immune system, inflammation, and circadian rhythms. In the present study, the synthetic RORα/γ agonist SR1078 stimulated the production and gene expression of FGF21 in C2C12 myotubes. FGF21, a member of the FGF family, plays an important role in the regulation of peripheral glucose tolerance and lipid metabolism while improving metabolic health. The mRNA expression and secretion of FGF21 were significantly weaker in RORα-silenced cells than in cells transfected with non-targeting control siRNA. SR1078 significantly up-regulated C/EBP homologous protein (CHOP), an established marker of ER stress, in a dose-dependent manner in C2C12 myotubes, while CHOP expression was decreased in RORα -silenced C2C12 cells, suggesting that RORα is involved in the regulation of FGF21 expression and stimulates ER stress in C2C12 myotubes. The naturally occurring compound baicalein up-regulated FGF21 expression and secretion in C2C12 myotubes. Additionally, the up-regulation of CHOP mRNA and protein expression was observed in C2C12 myotubes after the baicalein treatment. Furthermore, the knockdown of RORα prevented the augmentation of FGF21 and up-regulation of CHOP in response to baicalein in C2C12 cells. Collectively, these results suggest that baicalein stimulates the ER stress response and FGF21 expression through a RORα-dependent mechanism in C2C12 myotubes, and indicates the potential of baicalein as an effective anti-obesity therapy via its ability to enhance FGF21 production (Hirai et al., 2019). Other reports have shown that the hepatic expression of FGF21 is either regulated positively or negatively by glucocorticoid receptor (GR) (Patel et al., 2015), cAMP-responsive element-binding protein H (CREBH) (Lee et al., 2017), carbohydrate response element-binding protein (ChREBP) (Iizuka et al., 2009), PPARγ (Moyers et al., 2007; Wang et al., 2008), farnesoid X receptor (FXR) (Cyphert et al., 2012), and liver X receptor (LXR) (Archer et al., 2012; Uebanso et al., 2012) under various conditions. Extrahepatic tissues, such as skeletal muscle, white adipose, and brown adipose tissue regulate FGF21 via different transcription factors. Indeed, PPARγ activation increases FGF21 production in white adipose tissue where it acts as an autocrine or endocrine factor to improve insulin action (Dutchak et al., 2012). Brown adipose tissue (BAT), FGF21 is regulated by ATF2 (Hondares et al., 2011) while the skeletal muscle is controlled by ATF4 (Kim et al., 2013) and by the PI3K–AKT signaling pathway (Izumiya et al., 2008).

## FGF21 Downstream Signaling

The FGF21-dependent signaling downstream FGF receptors (FGFr) are extremely complicated and well debated. Its complexity is due to the fact that FGF21 is activated and released in different conditions such as energy stress, ER stress, mitochondrial dysfunction, and cold-stress. *In vitro* studies have failed to identify a direct interaction of FGF19 subfamily with their FGF receptor. This negative finding, and the very weak heparin binding affinity of these family members, implies that the FGF19 subfamily requires additional cofactors to stably bind the FGFRs in the target tissue (Gälman et al., 2008). The aging-suppressor gene Klotho and Beta-Klotho are indeed the essential co-receptors for the interaction and activation of FGFRs (Gälman et al., 2008). The complex FGF21-Klotho binds multiple FGFRs, including FGFR1c, -2c, -3c, and -4 both, *in vitro* and *in vivo* (Agrawal et al., 2018). However, the signaling downstream FGF receptors are tissue specific. In cultured adipocytes, FGF21 treatment activates Ras/Raf MAPK and the downstream effector ERK1 and ERK2 (Moyers et al., 2007; Ge et al., 2011). Transcriptomic analyses on adipocytes identified that ERK/MAPK signaling pathways are enriched after FGF21 administration *in vivo*, a second study showed that existing ERK1/2- independent pathways in mTOR act on FGF21 activating on adiponectin in adipose tissue *in vivo* (Minard et al., 2016). In liver, FGF21 positively controls the PI3K/AKT, insulin-like growth factor 1 (IGF-1) and mTOR pathways as well as triglyceride homeostasis, glucose uptake, amino acid transport, and energy expenditure (Muise et al., 2013). Moreover, FGF21 inhibits Growth Hormone (GH) action in hepatocytes resulting in STAT5 inhibition, decreased expression, secretion of IGF-I, and the reduction of body size (Inagaki et al., 2008). Finally, FGF21 also induces the expression of IGFBP1, an inhibitor of IGF1 and suppressor of cytokine signaling 2, which is a negative modulator of GH signaling, furthering blocking the GH-IGF1 axis in the liver. It has also been reported that FGF21 works on the central nervous system and dorsal vagal complex of the hindbrain to regulate circadian rhythm, which is important for the adaptive starvation response (Bookout et al., 2013).

## Serum Levels of FGF21 in Animal Models

In published studies, the serum FGF21 concentrations measured in chow-fed mice ranging from 0 to 3000 ng/mL (Badman et al., 2007; Fisher et al., 2010; Dutchak et al., 2012; Murata et al., 2013; Tezze et al., 2017; Jimenez et al., 2018). The difference in the reported FGF21 serum levels may be dependent on the mice strain, animal age, time of the day in which serum was collected, as well as the methodology used to quantify the serum levels. This complicates the interpretation of the data, since basal FGF21 concentration, as well as the increasing degree, may trigger different metabolic actions. As shown in Table 1, the fold increase of FGF21 serum in knockout /transgenic versus controls mice and in different experimental conditions are witness to a range from 2 to 100. These observations are particularly important considering that FGF21 is a molecule with a very short half-life that, in absence of specific stimuli, has been calculated from 1 to 2 h (Kharitonenkov et al., 2007; Xu et al., 2009; Hecht et al., 2012). This short half-life in the serum is due to enzymatic degradation and/or renal clearance. In fact, fibroblast activation protein α (FAP) was recently identified as the serine protease that cleaves and inactivates FGF21 (Zhen et al., 2016). Interestingly, FAP is also secreted in human muscle during exercise (Parmar et al., 2018). Therefore, because FGF21 is secreted from the kidney it has a short half-life and its activity is modulated by the presence of Klotho and FAP, simply monitoring serum levels may not be enough to claim a direct FGF21 action on targeted tissues without checking the activation of the pathway downstream FGFr.

**Table 1:** Serum level, absolute and normalize, of FGF21 regarding the transgenic mice, reported into the literature.

Transgenic mouse	Paper	Basal condition/control animals serum level (pg/mL)	Transgenic/virus serum level (pg/mL)	Fold increase compare to basal condition/control mouse
Virus-mediated FGF21 knock-down – liver specific	Badman et al., 2007		Not shown	
FGF21 total ko	Antonellis et al., 2016		Not shown	
FGF21 tg -liver specific	Inagaki et al., 2007		Not shown	
Virus-mediated FGF21 – liver specific	Jimenez et al., 2018	1000 (null-chow) 5000 (null-HFD)	Not shown (chow) 6000 – 25000 (HFD)	1–5 (HFD) (HFD)
Virus-mediated Fgf21 – muscle specific	Jimenez et al., 2018	3000 (fed)	45000(fed)	15 (fed)
atg7 ko – muscle specific	Kim et al., 2013	Detectable (fed) – 2000(fasted)	500(fed) – 4000(fasted)	Not clear (fed) – 2 (fasted)
Ucp1 tg – muscle specific	Keipert et al., 2013	1000(fed) – 2000(fasted)	4000(fed) – 5000(fasted)	4 (fed) – 2 (fasted)
4ebp1 tg – muscle specific	Tsai et al., 2015	1000 (fasted)	4000 (fasted)	4 (fasted)
Tsc1 ko – muscle specific	Guridi et al., 2015	500 (fed)	1500(fed)	3 (fed)
Opa1 ko – inducible muscle specific	Tezze et al., 2017	n.d. ( < 50 pg/mL)	4000 (fed)	100 (fed)
Opa1 ko – constitutive muscle specific	Rodríguez-Nuevo et al., 2018, p. 1	222 (fed)	544 (fed)	2,4
Opa1 ko – inducible muscle specific heterozygous	Pereira et al., 2017	1069 (fed)	11500 (fed)	10
Fundc1 ko – muscle specific	Fu et al., 2018	400	900 (HFD)	2,24


## FGF21 in Cardiac and Skeletal Muscle

**FIGURE 1:**
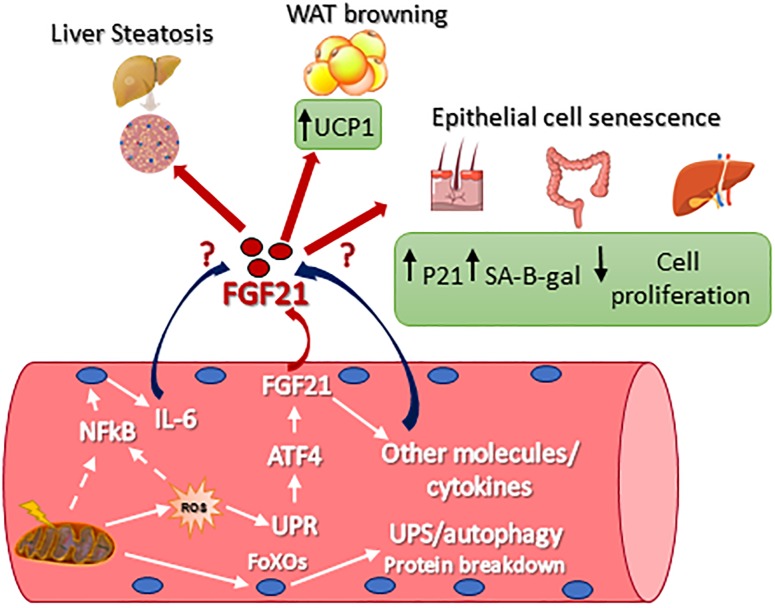
Signaling pathways that control FGF21 expression in skeletal muscle and FGF21-mediated metabolic effect on different tissues. UCP1, uncoupling protein 1; p21, Cdkn1a cyclin-dependent kinase inhibitor 1A; SA-B-gal, senescence-associated beta-galactosidase; Nf-kb, nuclear factor kappa-light-chain-enhancer of activated B cells; IL-6, interleukin 6; ROS, reactive oxygen species; ATF4, activating transcription factor 4; UPR, unfolded protein response; FoXO, Forkhead box O.

Originally, the heart was not considered to be an FGF21 source or target, primarily because β-Klotho mRNA is required to be co-receptors for cellular responsiveness to FGF21, and this was only found to be modestly expressed (Fon Tacer et al., 2010). However, recent studies have demonstrated that FGF21 plays a key role in cardiac remodeling. In fact, the heart expresses FGFR1, fibroblast growth factor receptor 1, β-klotho, as well as FGF21. Moreover, in rodents, it has been reported that FGF21 expression protects against pathologic cardiac hypertrophy, oxidative stress, and myocardial infarction (Planavila et al., 2013, 2015; Joki et al., 2015). In the heart, FGF21 acts in the manner of an autocrine and controls autophagy in obesity-induced cardiomyopathy (Rupérez et al., 2018). In skeletal muscle, FGF21 expression was found undetectable or to be limitedly expressed in basal conditions (Izumiya et al., 2008; Hojman et al., 2009; Pereira et al., 2017; Tezze et al., 2017; Rodríguez-Nuevo et al., 2018). However, several physiological and/or pathological conditions triggered FGF21 expression in muscle as well as showing a secretion into the blood. This was first demonstrated in muscle-specific AKT1 transgenic mice, where the activation of AKT1 brought a reduction of adipose tissue via FGF21 secretion (Izumiya et al., 2008). In Kim et al. (2013), it was found that autophagy inhibition, specifically in skeletal muscle, was protected from obesity and HFD because FGF21 was dramatically induced in muscles. As a consequence of the autophagic impairment muscles accumulate dysfunctional mitochondria, resulting in ER stress, UPR activation, and FGF21 induction via the transcription factor of ATF4. Similarly, muscle-specific deletion of Tuberous sclerosis 1 (TSC1) resulted in the activation of mTOR. At the same time FGF21 upregulation, via UPR, improved the insulin sensitivity, leading to an association with a leaner phenotype when compared with controls (Guridi et al., 2015). Consistent expression of constitutively active 4EBP1 in skeletal muscles caused FGF21 secretion into the circulation which then ameliorates lipid metabolism in WAT via PPARs (Tsai et al., 2015). Mild mitochondrial dysfunction is a consequent of UCP1, Uncoupling Protein 1, expression in skeletal muscle resulting in FGF21 expression and the diminution of the size of myofibers, but at the same time also improved the metabolic profile due to the browning of WAT (Keipert et al., 2013). We have now shown that mitochondrial dysfunction is due to OPA1 ablation in skeletal muscle and triggers a dramatic increase of muscle FGF21 transcript and serum levels (Tezze et al., 2017; Pereira et al., 2017; Rodríguez-Nuevo et al., 2018). This important FGF21 induction is mediated by mitochondrial-dependent oxidative stress that had caused UPR activation (Tezze et al., 2017; Rodríguez-Nuevo et al., 2018). ER stress, muscle atrophy, and FGF21 upregulation were found in several muscle-specific OPA1-deficient mice, but the phenotypes of these knockout mice were different. These discrepancies may be a consequence to the age of the animal in which OPA1 was deleted, and/or the degree of mitochondrial dysfunction, and/or the blood levels of FGF21. In Tezze et al. (2017) the 100-fold (Table 1) showed an increase of FGF21 blood levels after acute deletion of OPA1 mice at 5 months of age, causing several detrimental systemic effects. OPA1 inhibition induces important metabolic changes and a precocious aging phenotype of epithelial tissues, a systemic inflammatory response and a premature animal death (Figure 1). Importantly, FGF21 deletion in OPA1-null mice reverted almost all the effects linked to aging, while muscle and white adipose tissue loss were attenuated, but still present, in the double FGF21/OPA1 knockout mice. Interestingly, β klotho and FGFRs were upregulated in OPA1 deficient skeletal muscles when compared to the controls supporting the role of this pathway in muscle mass regulation (Tezze et al., 2017). Similar to Tezze et al. (2017) also Rodríguez-Nuevo et al. (2018) showed precocious mouse death and a systemic inflammatory response when OPA1 was deleted. In this work, the group of Zorzano used a myogenin-driven Cre transgenic mouse line to delete OPA1 in developing muscle. The finding that the phenotype of these knockout mice was not rescued by FGF21 inhibition is probably due to its reliance on unknown bioenergetics issues. In fact, the presence of mitochondrial dysfunction in growing muscles is too bioenergetically detrimental to be counteracted by concomitant FGF21 ablation. Alternatively, the secretory pattern induced by a mitochondrial defect in new-born mice is neither the same of adult muscle nor adequate to promote tissue senescence. Dale group has also generated an inducible muscle-specific knockout mouse that shows mild muscle loss and, surprisingly, several beneficial metabolic changes in terms of resistance to obesity when the animals were challenged with a high-fat diet. However, OPA1 deletion was induced in very young mice (4 weeks of age), and not in adult animals [12 weeks in Tezze et al. (2017)] and the reduction of OPA1 was partial (around 50–70%). This is probably due to the fact that stem cells muscles are still fused to myofibers, bringing the OPA1 gene inside the knockout fibers and causing the partial inhibition of OPA1 mildly affecting the mitochondrial function. In fact, mitochondrial respiratory complexes and supercomplexes, as well as mitochondrial DNA content and citrate synthase, were unaffected in these knockout mice. The partial OPA1 inhibition induced morphological changes (swelling) and minor alterations of respiration. This mild mitochondrial phenotype results in a minor level of FGF21 serum fold increase (Table 1). This phenotype differs from our animal model in which OPA1 deletion occurred in adult mice (3–5 months of age) and resulted in the decrease of mtDNA, respiratory complexes and supercomplexes content, complex activity, respiration, mitochondrial membrane potential and in an increase of ROS production. Altogether these mitochondrial changes resulted in a dramatic increase of FGF21 in muscle and serum (100 fold, Table 1), its range has been reported in patients with mitochondrial diseases (Morovat et al., 2017) and unhealthy aging (Conte et al., 2018). Therefore, the beneficial or detrimental effect of FGF21 is age, dose, time, and context/tissue-dependent.

## FGF21 in Humans, a Beneficial or Detrimental Molecule?

In healthy humans, a large variation level of serum FGF21 is shown between individuals, ranging from 5 pg/mL to 5 ng/mL (Gälman et al., 2008; Zhang et al., 2008; Li et al., 2009; Dushay et al., 2010; Fazeli et al., 2015). This finding is similar to the rodents’ data and further underlines a problem in the method of FGF21 quantification and/or in the variability in terms (Pedersen et al., 2003). For years the search for the stimulus that initiates and maintains the change of excitability or sensibility of the regulating centers in exercise has been progressing. For lack of more precise knowledge, it has been called the “work stimulus,” “the work factor,” or “the exercise factor.” In other terms, one big challenge for muscle and exercise physiologists has been to determine how muscles signal to central and peripheral organs. Here, we discuss the possibility that interleukin-6 (IL-6) could mediate some of the health beneficial effects of exercise. In resting muscle, the IL-6 gene is silent, but it is rapidly activated by contractions. The transcription rate is very fast and the fold changes of IL-6 mRNA is marked. IL-6 is released from working muscles into the circulation in high amounts. The IL-6 production is modulated by the glycogen content in muscles, and IL-6 thus works as an energy sensor. IL-6 exerts its effect on adipose tissue, inducing lipolysis and gene transcription in abdominal subcutaneous fat and increases whole body lipid oxidation. Furthermore, IL-6 inhibits low-grade TNF-alpha-production and may thereby inhibit TNF-alpha-induced insulin resistance and atherosclerosis development. We propose that IL-6 and other cytokines, which are produced and released by skeletal muscles, exerting their effects in other organs of the body, should be named “myokines” (Pedersen et al., 2003). Of the patient’s age, time of the day when the blood is collected, and nutritional state of the patient (fed or fasted) that may affect FGF21 secretion or turnover. The *Fgf21* gene under basal conditions is considered to be mainly expressed in the liver, and to a much lesser extent, also in the brain (Kharitonenkov and Adams, 2014) and in the pancreas (Fon Tacer et al., 2010). Similar to rodents, the PPARα response element was identified in the human FGF21 promoters meaning that they may regulate FGF21 expression (Lundåsen et al., 2007). However, there are other tissues contributing to the FGF21 blood levels in physio-pathological conditions. In skeletal muscle, FGF21 is expressed in response to insulin stimulation, suggesting that FGF21 is an insulin-regulated myokine (Hojman et al., 2009) and an association between chronic hyperinsulinemia and levels of FGF21 were found in humans (Hojman et al., 2009). Fazeli et al. (2015) showed that FGF21 works as a fasting-induced hormone for the adaptive response to starvation and for the utilization of fuel derived from tissues breakdown. Others claimed that serum levels of FGF21 are closely related to adiposity, lipid metabolism, and are used as a biomarker of liver injury but not of insulin secretion and sensitivity (Li et al., 2009). Despite the beneficial action of FGF21 in rodents, the literature in humans is not homogeneous and is supportive of some detrimental effects. For instance, FGF21 serum levels are increased in patients with mutations of mitochondrial DNA in skeletal muscles but not when similar mutations accumulate in other organs. Due to this specificity to mitochondrial myopathies, FGF21 was proposed as a biomarker of mitochondrial dysfunction in skeletal muscles and used as a diagnostic test for these inherited disorders (Suomalainen et al., 2011, p. 21). In other conditions FG21 serum levels have been used as a predictor of disease progression. For instance, higher circulating FGF21 levels were associated with a high mortality rate in end-stage renal disease patients (Kohara et al., 2017). It was also reported in patients with diastolic dysfunction of heart failure who have preserved ejection fraction (Chou et al., 2016), and in those who are serum FGF21 level had been found to be an independent predictor of coronary heart disease (Lee et al., 2017). Moreover, circulating FGF21 levels are elevated in various metabolic disease states, such as obesity, insulin resistance, and type 2 diabetes mellitus (Zhang et al., 2008; Chavez et al., 2009). Interestingly, patients with type 2 diabetes also have elevated circulating levels of FAP, and this is associated with a diminished ratio of bioactive to total FGF21 in response to an oral glucose tolerance test (Samms et al., 2017). The increase of plasma FGF21 levels correlated with the severity of whole-body mass (primarily muscle) and hepatic insulin resistance (Chavez et al., 2009). Unexpectedly, in obese humans, the levels of FGF21 did not change after chronic exercise (Besse-Patin et al., 2014) despite the well-known beneficial effect of physical activity. This could be related to the level of the myokine FAP, which is known to increase during exercise (Parmar et al., 2018). Increased FAP could weaken the ratio of bioactive FGF21 in obesity as well as in TDM2 patients. Moreover, we found that FGF21 serum levels positively and significantly correlated with age (Tezze et al., 2017; Conte et al., 2018). Importantly, the presence of high FGF21 serum levels in association with GDF15 and humanin, which are two other markers of mitochondrial function, had been found to be a predictor of morbidity and mortality (Conte et al., 2018).

## Final Considerations

The understanding that the control of whole-body metabolism has been greatly advanced in the last years. Impacting these factors are the different molecules which are secreted by skeletal muscles in different conditions. In particularly, FGF21 emerged as an important myokine with several metabolic effects but whether its action is beneficial or detrimental in physiological or pathological conditions are still unclear, especially in humans. There are several issues that should be addressed and clarified soon. The most problematic and urgent one is related to the great variability in the measurements of blood levels between different studies, both in mice and humans. Therefore, a standardization of the procedure for FGF21 measurement is required to understand whether a threshold of serum concentrations is a determinant for its different actions. Nevertheless, we have a clear picture of the different tissues source and the targets that are involved in FGF21 effects. We also know the conditions that trigger FGF21 expression in skeletal muscles (e.g., mitochondrial dysfunction and ER stress). What is still unclear is the relationship between FGF21-mediated metabolic changes and the contribution of these alterations to disease progression/onset. Moreover, the molecules that synergize, enhance or counteract FGF21-action are still unknown. Our recent findings are that high versus low serum levels correlate with mortality in elderly people, underline the concept that FGF21 actions are dose-dependent. Therefore, first, it is necessary to better address which concentration, or fold increase in plasma, is the most relevant for obtaining a beneficial or detrimental effect. Second, it is important to dissect the other factors (e.g., cytokines, metabolites) that may modulate FGF21 action in target tissues and consequently elicit a positive (healthy) or negative (unhealthy) effect. Finally, the downstream signaling pathway should be better dissected in beneficial or detrimental action. In conclusion, FGF21 belongs to a promising class of cytokines that are induced in response to stress and that can be used as a drug, drug target, or through a biomarker, depending on the physio-pathological context. All these findings will become clear when FGF21 will be used as a therapeutic molecule, exploiting the beneficial effects of FGF21 for treating metabolic disease or when it will be blocked to ameliorate disease progression and the onset of disease.

## Author Contributions

CT and MS wrote the manuscript. VR contributed to the discussion.

## Conflict of Interest Statement

The authors declare that the research was conducted in the absence of any commercial or financial relationships that could be construed as a potential conflict of interest.
